# A 12-Week Cycling Training Regimen Improves Gait and Executive Functions Concomitantly in People with Parkinson’s Disease

**DOI:** 10.3389/fnhum.2016.00690

**Published:** 2017-01-12

**Authors:** Alexandra Nadeau, Ovidiu Lungu, Catherine Duchesne, Marie-Ève Robillard, Arnaud Bore, Florian Bobeuf, Réjean Plamondon, Anne-Louise Lafontaine, Freja Gheysen, Louis Bherer, Julien Doyon

**Affiliations:** ^1^Research Center of the University Institute of Geriatrics of MontrealMontreal, QC, Canada; ^2^Functional Neuroimaging UnitMontreal, QC, Canada; ^3^Department of Psychology, University of MontrealMontreal, QC, Canada; ^4^Department of Psychiatry, University of MontrealMontreal, QC, Canada; ^5^Centre for Research in Aging, Donald Berman Maimonides Geriatric CentreMontreal, QC, Canada; ^6^PERFORM Centre, Concordia UniversityMontreal, QC, Canada; ^7^Department of Electrical Engineering, Polytechnique MontrealMontreal, QC, Canada; ^8^McGill Movement Disorder Clinic, McGill UniversityMontreal, QC, Canada; ^9^Department of Movement and Sport Sciences, Ghent UniversityGhent, Belgium; ^10^Department of Medicine, University of MontrealMontreal, QC, Canada; ^11^Montreal Heart InstituteMontreal, QC, Canada

**Keywords:** Parkinson’s disease, exercise, gait, aerobic, stationary bicycle

## Abstract

**Background:** There is increasing evidence that executive functions and attention are associated with gait and balance, and that this link is especially prominent in older individuals or those who are aﬄicted by neurodegenerative diseases that affect cognition and/or motor functions. People with Parkinson’s disease (PD) often present gait disturbances, which can be reduced when PD patients engage in different types of physical exercise (PE), such as walking on a treadmill. Similarly, PE has also been found to improve executive functions in this population. Yet, no exercise intervention investigated simultaneously gait and non-motor symptoms (executive functions, motor learning) in PD patients.

**Objective:** To assess the impact of aerobic exercise training (AET) using a stationary bicycle on a set of gait parameters (walking speed, cadence, step length, step width, single and double support time, as well as variability of step length, step width and double support time) and executive functions (cognitive inhibition and flexibility) in sedentary PD patients and healthy controls.

**Methods:** Two groups, 19 PD patients (Hoehn and Yahr ≤2) and 20 healthy adults, matched on age and sedentary level, followed a 3-month stationary bicycle AET regimen.

**Results:** Aerobic capacity, as well as performance of motor learning and on cognitive inhibition, increased significantly in both groups after the training regimen, but only PD patients improved their walking speed and cadence (all *p* < 0.05; with no change in the step length). Moreover, in PD patients, training-related improvements in aerobic capacity correlated positively with improvements in walking speed (*r* = 0.461, *p* < 0.05).

**Conclusion:** AET using stationary bicycle can independently improve gait and cognitive inhibition in sedentary PD patients. Given that increases in walking speed were obtained through increases in cadence, with no change in step length, our findings suggest that gait improvements are specific to the type of motor activity practiced during exercise (i.e., pedaling). In contrast, the improvements seen in cognitive inhibition were, most likely, not specific to the type of training and they could be due to indirect action mechanisms (i.e., improvement of cardiovascular capacity). These results are also relevant for the development of targeted AET interventions to improve functional autonomy in PD patients.

## Introduction

Parkinson’s disease (PD) is a neurodegenerative pathology characterized by progressive motor symptoms, including gait modifications leading to balance instability (Bello et al., 2010). Patients can also develop several non-motor complications, such as depression, sleep disturbances and cognitive impairments like executive dysfunctions (Speelman et al., 2011) and deficits in procedural learning (Clark et al., 2014; Ruitenberg et al., 2015). Despite advances in pharmacological agents and surgical procedures that could be employed in PD patients to alleviate the primary motor signs of the disease, these treatment options often fail to improve the whole range of symptoms observed in PD and side effects are common (Bloem et al., 2004). Recently, exercise has been proposed as an adjuvant therapy that may help in alleviating multiple symptoms, but very little is know about the impact and the mechanisms of such alternatives.

Despite the fact that the link between gait and cognitive functions, especially executive functions, is well documented in aging research (Springer et al., 2006; Yogev-Seligmann et al., 2008; Liu-Ambrose et al., 2010; Martin et al., 2013), to date, only a few studies have investigated the same relationship in PD patients. In contrast, there is growing evidence documenting the association between standing balance and gait initiation with cognition in PD population, but in a dual-tasking context (Fernandes et al., 2015, 2016). The latter have shown that some cognitive processes, such as executive functions, processing speed and semantic fluency (Smulders et al., 2013; Stegemoller et al., 2014) are associated with some gait parameters and functional mobility in PD. In addition, several studies have shown independently that non-pharmacological treatment approaches, such as physical exercise (PE), do improve various gait parameters in PD (Mehrholz et al., 2010; Li et al., 2012; Shu et al., 2014; Arcolin et al., 2015), on the one hand, and executive functions, on the other hand (Tanaka et al., 2009). Yet, evidence that this type of intervention can simultaneously improve motor (such gait) and non-motor symptoms (executive functions, motor learning) in PD is non-existent and the mechanisms by which training produces changes in both of these components remain unknown.

We have recently reported that an aerobic exercise training (AET) regimen using a stationary bicycle is not only safe for PD patients in early stages of the disease, but that it has also improved aerobic capacity as well as cognitive inhibition and motor sequence learning (MSL; Duchesne et al., 2015). In the current study, our main objective was to assess the effects of an AET regimen using stationary bicycling on gait parameters in sedentary people with PD (not reported in the previous study). As a second objective, we set out to compare these effects to those observed in healthy adults (HA) in order to determine whether this type of intervention has a different impact depending on the participant’s health status. In addition, as a third objective, we intended to evaluate the associations between exercise-related changes in gait (not reported in the previous study) with those seen in cardiovascular capacity, executive functions and motor sequence capacity (Duchesne et al., 2015). We hypothesized that: (1) bicycle training would improve gait parameters in all participants (especially speed and cadence based on the specificity of the bicycle training), but especially those diagnosed with PD, (2) such improvements in gait parameters would correlate with other AET-related improvements, such as cardiovascular capacity, cognitive inhibition and the capacity to learn a new sequence of movements, and that (3) these relations would be moderated by disease.

## Materials and Methods

### Participants

In order to be eligible for the study, all participants (HA and those with PD) had to be right-handed, sedentary, and aged between 40 and 80 years old. They were screened for the presence of possible dementia score between 24 and 30 needed on the Mini Mental State Evaluation (Folstein et al., 1975) or on the Montreal Cognitive Assessment (Marinus et al., 2011) and appropriateness for testing in an MRI environment (e.g., no metallic implants that could interfere with testing, no claustrophobia). The Physical Activity Readiness Questionnaire (PAR-Q) was used to verify the participant’s safety in participating in a physical program. Exclusion criteria included other neurological disorders, and comorbidities likely to affect gait, smoking or heart diseases. Importantly, HA were matched with PD patients with respect to sex distribution, age, education as well as cognitive and fitness levels. PD patients had to be classified as stage 1 or 2 according to Hoehn and Yahr’s scale based upon evaluation of a certified neurologist (A-LL). Participants who were under medication continued their treatment all throughout the study (testing and training). This study was carried out in accordance with the recommendations of the research ethics committees’ guidelines of the Research Center of the University Institute of Geriatrics of Montreal, which approved the protocol. Written and informed consent was obtained from each participant in this study. Demographic characteristics of the samples are presented in **Table 1**.

**Table 1:** Demographic data.

Characteristics	HA	PD	Group differences
Demographic information			
Age (years)	64 ± 8.19	59 ± 7.11	*p* = 0.06
Ratio men/women	8/12	13/6	*p* = 0.07
Education (years)	15.7 ± 2.36	15.05 ± 2.78	*p* = 0.43
Overall cognitive level			
Cognition (MMSE/MoCA)	29.18 ± 1.25	28.4 ± 1.34	*p* = 0.28
	29.56 ± 1.51	27.21 ± 1.85	*p* = 0.08
Psychological well-being			
Depression (BDI)	4.8 ± 4.5	10.5 ± 8.3	*p* < 0.01
Anxiety (BAI)	2.1 ± 2.7	8.6 ± 9.4	*p* < 0.01
Executive functions			
Inhibition (Stroop, in s)	115.4 ± 4.7	128.5 ± 6.7	*p* = 0.12
Flexibility (TMT, in s)	75.0 ± 6.4	85.5 ± 10.5	*p* = 0.39
Clinical variables			
UPDRS III	N/A	21.84 ± 6.16	N/A
Duration of disease (years)	N/A	8.1 ± 9.12	N/A
Hoehn and Yahr	N/A	2.1 ± 0.2	N/A
Gait parameters			
Walking speed (m/s)	1.14 ± 0.03	1.10 ± 0.04	0.386
Cadence (steps/min)	115.49 ± 2.00	110.05 ± 2.72	0.116
Step length (m)	0.593 ± 0.064	0.598 ± 0.080	0.833
Step width (m)	0.207 ± 0.032	0.215 ± 0.035	0.455
Single support time (s)	0.401 ± 0.006	0.417 ± 0.009	0.154
Double support time (s)	0.12 ± 0.01	0.13 ± 0.01	0.339
Step length variability	0.012 ± 0.009	0.011 ± 0.006	0.737
Step width variability	0.009 ± 0.007	0.011 ± 0.007	0.593
Double support time variability	0.009 ± 0.002	0.007 ± 0.001	0.905

*Means ± SD. HA, healthy adults; PD, Parkinson’s diseases individuals; N/A, non-applicable; s, seconds; m, meters; min, minute.*

### Exercise Intervention Protocol

Prior to engaging in the training regimen, all participants were cleared by a physician, who analyzed the electrocardiogram (ECG) at rest in order to rule out any cardiac anomalies. The aerobic exercise intervention was designed to improve cardiorespiratory fitness with an exercise intensity prescription based on each participant’s maximal aerobic power output achieved at maximum volume of oxygen (VO_2_ peak) uptake assessed on the pre-test day (ACMS, 2006). Recumbent bicycles were used to train participants. Duration of the exercise program started at 20 min and 60% of intensity per session, and was then increased by steps of 5 min and 5% of intensity every week, until participants reached 40 min of training at 80% intensity. Bike speed was maintained at 60 revolutions per minute (RPM). As such, to achieve the desired bike resistance power and adjust intensity level (if needed), the work intensity was based on power output (Watt), controlling for participant’s heart rate. In addition, rate of perceived exertion (Borg scale; Borg, 2012) was assessed during each training session. The program lasted 12 weeks, with three training sessions per week. A participation rate equivalent to 75% of the sessions was achieved by each participant included in the data analyzes. Trained kinesiologists supervised all sessions.

### Assessments

Participants were evaluated on a variety of outcome measures before the intervention and immediately after completion of the 3-month exercise program.

Lower limb capacities were assessed with the GaitMat II (E.Q. Inc., Chalfont, PA, USA; Barker et al., 2006). The GaitMat II consists of a 7.8-m long walkway and its computer software, which controls the mat sensors and calculates different metrics of gait. The mat is also equipped with initial and final 1 m inactive sections that allow acceleration and deceleration of the participant locomotion. One trial consisted of participants walking the full length of the Mat at their self-selected walking speed. After a practice of two trials, participants completed four more trials for data collection and measurements (walking speed, cadence, step length, step width, single and double support time, as well as variability of step length, step width and double support time).

To evaluate the patient’s mood, the Beck Depression Inventory (BDI; Beck et al., 1961) and the Beck Anxiety Inventory (BAI; Beck et al., 1988) were used. Also, executive functions were assessed, precisely cognitive inhibition and flexibility. Participants’ inhibitory aptitude was assessed using a version of the Stroop test with three different conditions (naming, reading, and interference). Each condition contained 100 stimuli (i.e., words, colored rectangles, words in colors) printed on a 21.5 cm × 28 cm sheet of paper. In the reading condition, participants had to read the words (red, green, blue, and yellow) printed in black. In the naming condition, subjects had to name of the rectangles. In the third condition (interference), individuals needed to name the color of the ink in which the words were written. In the latter condition, the meaning of each word had to be ignored, as it was incongruent with the color to name (i.e., the word “blue” written in yellow). The trail Making Test (TMT) was used to assess subjects’ flexibility functions. The first part of the test (TMT A) included numbers from 1 to 25, circled and written on a 21.5 cm × 28 cm sheet of paper. Participants were asked to connect with a pencil, as fast as possible, the numbers in numerical order. In contrast, the second part (TMT B) included numbers from 1 to 13 and letters from A to L. Subjects were asked to connect, as fast as possible, a number followed by a letter in numerical and alphabetic order, respectively (i.e., 1-A-2-B-etc.). The participants’ capacity in MSL was evaluated during a functional magnetic resonance imaging session, where they had to perform an implicit serial reaction time task (Nissen and Bullemer, 1987). More details of the complete fitness, psychological, neuropsychological and motor learning evaluations can be found in our previous study by Duchesne et al. (2015).

### Statistical Analysis

A repeated model ANOVA was used to test the effect of AET on primary and secondary outcomes in PD participants. In addition, a mixed model ANCOVA was carried out to assess group differences pre-post AET, as well as the effect of training within each group and group differences at baseline and after AET for all gait parameters. BDI scores and age were used as covariates for all analyses to account for group differences in sentiments of depression and age, two factors that may impact gait parameters such walking speed (Rochester et al., 2008). In order to account for the effect of multiple comparisons, the statistical significance was adjusted using the Bonferroni method. All results were expressed as means ± standard deviations for descriptive statistics. Pearson linear correlations between walking speed, cadence and step length with aerobic capacity, executive functions (inhibition and flexibility) and MSL (performance and learning scores) were tested to figure out if there is a link between gait and these factors among PD participants only. We then employed Hayes’s (2009) free add-on SPSS macro to test whether the disease (present/absent) moderated the relationship between gait parameters and other variables of interest, i.e., if the relation between variables in PD is in the same direction than the group of reference. Training-related changes in cognitive scores, MSL, and aerobic capacity constituted the independent variables in the moderation model, while gait parameters that changed significantly as a result of training corresponded to the outcome variable. Analyses were conducted using SPSS 21.0 (IBM, Armonk, NY, USA). The level of statistical significance for all tests was set at *p* < 0.05.

## Results

Forty-four participants (21 PD patients and 23 HA) were deemed eligible to enroll in the study after the completion of the first evaluation. However, between the evaluation and the beginning of the exercise program, two HA decided to withdraw from the project for personal reasons. One PD participant and one HA were excluded after the beginning of the training regimen for health security reasons. Only one PD patient was excluded from analysis after AET regimen completion because of extreme results on several outcomes, even if this person respected all inclusion criteria. In the end, a total of 39 persons (19 PD patients and 20 HA) were analyzed. All demographic characteristics and initial values of the study participants are described in **Table 1**. There was no difference between groups for any of the gait parameters at baseline.

**FIGURE 1:**
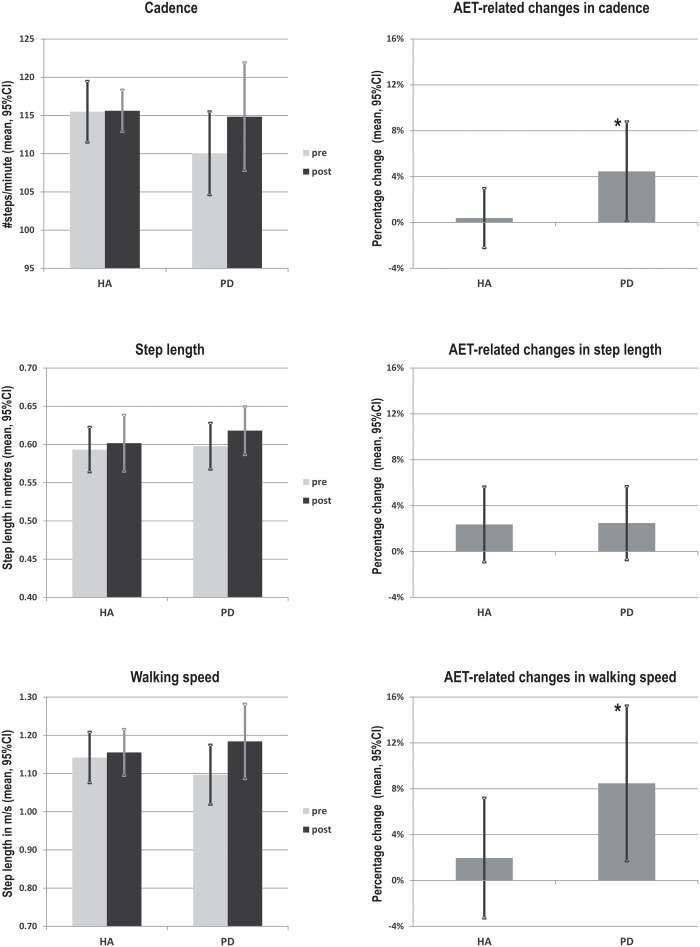
**AET-related changes in walking speed, cadence and step length in PD patients and healthy adults.** ^∗^Indicate statistically significant differences pre- to post- training (*p* < 0.05). HA, healthy adults; PD, Parkinson’s diseases patients.

Following the 12-week AET, repeated measures ANOVA indicated that PD participants showed significant improvements for the walking speed (*F*_1,18_ = 6.154, *p* < 0.05), the step length (*F*_1,18_ = 5.828, *p* < 0.05) and the single support time (*F*_1,18_ = 4.771, *p* < 0.05), with a trend for cadence (*F*_1,18_ = 4.211, *p* = 0.055). When using the mixed ANCOVA model, the effect observed in PD for walking speed and single support time remained the same while the one obtained for step length disappeared (**Figure 1**). However, the trend observed for the cadence became significant when using covariates such the age and the BDI (*p* < 0.05). In addition, the groups did not differ significantly neither in pre- or post-comparisons, nor in regards to AET-related changes. All other gait parameters did not change significantly following the aerobic training (*p*s > 0.05) (**Table 2**).

**Table 2:** Spatiotemporal gait parameters during self-selected speed condition.

Gait variables	HC		PD		Statistical significance for the independent variables		
	Pre-AET	Post-AET	Pre-AET	Post-AET	AET	Group	Interaction
Walking speed (m/s)	1.14 ± 0.03	1.16 ± 0.03	1.10 ± 0.04	1.18 ± 0.05^†^	*p* = 0.28	*p* = 0.91	*p* = 0.27
Cadence (steps/min)	115.49 ± 2.00	115.61 ± 1.37	110.05 ± 2.72	114.84 ± 3.53^†^	*p* = 0.15	*p* = 0.70	*p* = 0.10
Step length (m)	0.593 ± 0.064	0.602 ± 0.066	0.598 ± 0.08	0.618 ± 0.069	*p* = 0.71	*p* = 0.73	*p* = 0.98
Step width (m)	0.207 ± 0.032	0.213 ± 0.028	0.215 ± 0.035	0.222 ± 0.037	*p* = 0.381	*p* = 0.727	*p* = 0.962
Single support time (s)	0.401 ± 0.006	0.398 ± 0.004	0.417 ± 0.009	0.384 ± 0.015^†^	*p* < 0.05^*^	*p* = 0.83	*p* < 0.05^*^
Double support time (s)	0.120 ± 0.010	0.117 ± 0.004	0.130 ± 0.010	0.136 ± 0.013	*p* = 0.25	*p* = 0.24	p = 0.21
Step length variability	0.52 ± 0.01	0.52 ± 0.01	0.55 ± 0.01	0.53 ± 0.02	*p* = 0.15	*p* = 0.41	*p* = 0.17
Step width variability	0.010 ± 0.007	0.011 ± 0.008	0.011 ± 0.007	0.008 ± 0.010	*p* = 0.166	*p* = 0.983	*p* = 0.509
Double support time variability	0.009 ± 0.010	0.006 ± 0.003	0.007 ± 0.004	0.033 ± 0.077	*p* = 0.417	*p* = 0.160	*p* = 0.087

*No significant differences between groups at baseline were found. Means ± SD. ^†^A significant within-group difference from baseline. HA, Healthy adults; PD, Parkinson’s disease patients; m, meters; s, second(s); min, minute; AET, aerobic exercise training. Bolded terms emphasize statistical differences.*

Significant between-sessions differences were found in both groups for outcomes related to aerobic capacity (VO_2_ peak), MSL capacity and cognitive inhibition (all *p* < 0.05), indicating that the training improved participants’ fitness, procedural learning and cognitive inhibition, regardless of the health status. Given that these results were analyzed in detail and reported elsewhere (Duchesne et al., 2015), they are presented here only for reader’s convenience in the Supplementary material (Supplementary Figure S1). However, these data were used to test the correlation with gait parameters among PD participants. We observed a significant association only between the walking speed at the post-test and the aerobic capacity after the AET (*r* = 0.461, *p* < 0.05, *N* = 19). No correlation was observed between other gait parameters and cognition or MSL.

**FIGURE 2:**
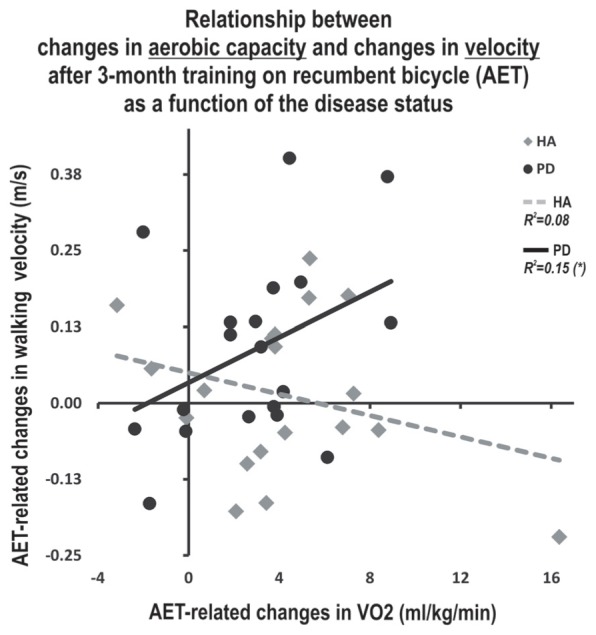
**Moderating effect of the disease on the relationship between AET-related changes in walking speed and aerobic capacity.** ^∗^Indicate statistically significant differences pre- to post- training (*p* < 0.05). HA, healthy adults; PD, Parkinson’s disease; AET, aerobic exercise training; m, meters, sec, second, ml: milliliters, kg, kilogram, min, minute.

A multiple regression model was then employed to investigate whether the association between pre-post change in walking speed and change in fitness depended upon the presence of disease (i.e., moderation). The relationship between change in fitness and change in walking speed was significantly moderated by the presence of the disease (*R*^2^ increase due to the interaction: *F*_1,32_ = 4.34, *p* < 0.05; conditional effect of change in fitness on change in walking speed: HA *t* = -1.08, *p* = 0.29, PD *t* = 1.79, *p* = 0.08). Specifically, patients with PD who increased their cardiorespiratory capacities the most also showed the best improvement in walking speed (**Figure 2**). By contrast, there was no significant relationship between these variables in HA (HA group: β = -0.162, *p* = 0.505; PD group: β = 0.596, *p* = 0.027). Also, no relationship between gait parameters and motor skill learning or executive functions was found alone or when investigating the moderation of these relationships by the presence of the disease.

## Discussion

In the current study, we investigated the effects of an AET regimen using stationary bicycling on gait parameters in sedentary HA and in PD patients. As reported previously by our group (Duchesne et al., 2015), such training regimen improved cardiovascular capacity, executive functions and motor learning capacities in both groups. Here, we report that AET also had a significant positive impact on cadence and walking speed in the PD group. Moreover, the presence of the disease mediated the relationship between aerobic capacity and walking speed, as the improvement in fitness correlated positively with that in walking speed of PD patients only. Contrary to our expectations, we did not find significant relationships between AET-related changes in gait parameters and cognition.

Importantly and as predicted, however, the present study yielded significant increases in walking speed and cadence in the PD group. The latter findings are consistent with previous studies indicating that 4–12 weeks of treadmill training improved walking speed, step length and step-to-step variability (Herman et al., 2009). This is also in accordance with other reports that resistance training, tai chi and physical therapy lead to improvements in walking speed and step length (Pellecchia et al., 2004; Li et al., 2012). Until now, studies on stationary bicycle training used forced exercise paradigm and observed improvements of dexterity, tremor and bradykinesia (Burini et al., 2006; Ridgel et al., 2009; Alberts et al., 2011). Finally, the fact that we did not find significant improvements in step length in PD patients, but observed significant increases in walking speed and cadence, may be due to the nature of our AET program. Indeed, the pedaling rhythm during exercising has a built-in cadence, and thus, it is expected that training-specific effect of bicycling would be more pronounced in terms of cadence, as compared to other gait parameters. This is similar to the mechanism by which treadmill training will impact more step length and walking speed, rather than other gait variables (Fisher et al., 2008; Herman et al., 2009).

Contrary to our expectations, there was no significant relationship between changes in PD participants’ executive functions, MSL, cardiovascular capacity and gait parameters. We had hypothesized that we would find a positive and significant relation between executive functions and gait in the PD group, mostly because previous evidence suggested that some elements of cognition such as working memory and attention capacities, were associated with gait abnormalities in PD (Smulders et al., 2013). Our hypothesis was based on the fact that in a recent study, Sohmiya et al. (2012) found significant correlation between frontal assessment battery scores and changes in gait following physical therapy (Sohmiya et al., 2012). Yet it is important to note that in that study, PD patients with high, but not low, executive functioning scores improved walking speed, stride and step length after physical training. Furthermore, unlike in our sample, PD participants in these studies were in more advanced stages of the disease. Thus this suggests that the relationship between gait and executive functions may be more evident as the disease progresses, hence possibly explaining why we did not observe any relation between gait parameters and other cognitive and learning functions. The fact that AET improved certain gait parameters and cognitive functions, but that these changes did not correlate with each other suggest that independent action mechanisms underlie this therapeutic improvements, at least at this stage of the disease.

We used moderation analyses to investigate the extent to which age influenced the relationship between changes in participants’ fitness levels as well as their executive functioning and motor learning capacities on the one hand, and gait parameters, on the other hand. Using such an approach, we found a significant moderation effect for the disease variable only, regarding the relationship between AET-related improvements in aerobic capacity and those in walking speed. It has previously been suggested that motor abilities in the PD population, such as gait, could be affected by various health conditions. For example, a decrease in cardiorespiratory capacity has been shown to affect walking speed (Skidmore, 2008). Therefore, our results, especially the positive correlation between improvements in VO_2_ peak and those in walking speed in PD patients only, seem to support this hypothesis. In addition, they suggest that motor abilities may be improved in PD via non-pharmacological means, such as aerobic PE.

The main objective of our study was to assess of AET in PD patients. We used the HA group to explore the possibility that AET may have a differential impact as a function of disease. However, we found no significant differences between these two groups in regards to any of the primary and secondary outcomes, neither in pre-, post- or AET-related changes. This is in contrast with the few studies that compared gait parameters in these two populations (Frenkel-Toledo et al., 2005; Bello et al., 2008). Thus, although conjectural, the reason why we did not observe any group differences may be that we recruited PD patients that were in the early phase of the disease (i.e., Hoehn and Yahr stage 1 or 2), compared to previous reports which included patients in more advanced stages (Frenkel-Toledo et al., 2005; Bello et al., 2008).

Several mechanisms have been proposed to explain physical training-related improvements similar to those seen in the present study. Some of these include direct effects on the central nervous system based upon an optimisation of the medication intake by easing its absorption (Speelman et al., 2011) or through increased corticomotor excitability (Fisher et al., 2008) and dopaminergic neurotransmission (Petzinger et al., 2010). Other proposed mechanisms have been more indirect and include increased cortical vascularisation, synaptic plasticity and neurogenesis (Speelman et al., 2011). These processes, which could be mediated by neurotrophic factors, would lead to structural and functional brain changes. Although the present study does not allow identifying the mechanism(s) that could explain the effects of AET on gait measures reported here, one can nevertheless assume that some brain structural and functional changes could be at the origin of such clinical outcomes. For example, in rodents, regional gray matter volume in a region equivalent to supplementary motor area (SMA) in humans has shown to be correlated positively with the total distance run by the animal following 7 days of exercise (Sumiyoshi et al., 2014). Similar increases, in both white and gray matter, have been reported in a group of older sedentary human adults after participation in a 6-month aerobic training regimen (Colcombe et al., 2006). Furthermore, a recent study using resting state functional magnetic resonance imaging found significant changes in activity in sensorimotor areas in a group of young individuals after 20 min of aerobic exercise (Rajab et al., 2014), while a couple of studies demonstrated that functional brain activity in motor areas increased proportional to the movement rate on a pedaling task executed during scanning (Mehta et al., 2012). Finally, increased SMA activation was observed during motor imagery of locomotor-related tasks (Malouin et al., 2003) as well as during real locomotion as measured by electrophysiological studies showing SMA modulation during walking (Petersen et al., 2012; Wagner et al., 2012, 2014; Seeber et al., 2015). Thus despite the scarcity of studies assessing cerebral structural and functional changes in relation to gait parameters and aerobic training in humans, the above mentioned studies provide basic evidence that AET can have a direct impact on the brain. Given the lack of neuroimaging studies looking at the effect of PE in PD, further investigations are needed to directly assess these mechanisms in PD patients using neuroimaging techniques.

An issue that merits discussion would be the role of medication. There is evidence that medication itself may have a differential effect on movement rate and amplitude, as demonstrated by past research (Espay et al., 2009, 2010, 2011; Stegemöller et al., 2009; Teo et al., 2013, 2014). In the current study, we believed that medication did not play a major role influencing the outcomes for PD patients. The reason is that patients were always on their medication during both pre and post evaluations, their medication did not change and more importantly, assessments always took place at the same time of the day.

Although our findings help increasing our knowledge base about the effects of AET on gait, the current study has some limitations. A limitation of the present study was the lack of an additional training condition controlling for the type and intensity of exercise that PD patients performed over the 3-month period. However, despite this shortcoming, we believe that the results of the current study are theoretically and clinically relevant as they suggest that the use of AET using stationary bicycle can have a beneficial impact in persons suffering from PD. Another limitation is the fact that we only assessed two cognitive functions, inhibition and flexibility, which are the most commonly used in the PD literature. Therefore, one cannot exclude the possibility that other cognitive domains may show correlations with gait parameters following physical training.

From a clinical perspective, the use of high-intensity exercise is now a common rehabilitation method in PD. It is important to note that the AET effects in the current study were observed after a moderate to a high intensity exercise regimen (half of the program was performed at 80% of maximal intensity). Thus, with its stable and comfortable sitting posture, our results suggest that AET with a stationary bicycle is not only a viable, but also a safe training procedure for PD patients in stage 1 and 2 of the disease. Further studies are still needed in order to assess the safety and feasibility of a training regimen on stationary bicycle in patients in more advanced stages of the disease who are showing greater physical limitations such as balance impairments, as well as to evaluate the long-term benefits of this training method. Yet, despite such limitations, our study shows that stationary bicycle can be successfully used to improve gait functions in PD patients. The main contribution of the current study thus stems from the fact that our findings are showing AET-related improvements in a gait parameter (walking speed) is crucial for the daily functioning of sedentary patients who are in the initial stages of the disease. Therefore, we believe that this result is another step closer to developing a reliable strategy to stimulate an active lifestyle in patients with PD, taking into account safety issues and each patient’s individual capacities.

## Ethics Statement

RNQ- Research Ethics Mixed Committee. The protocol was approved by a research committee to ensure the full security of the participants. The consent form was read with participants before to obtain their agreement to participate.

## Author Contributions

Research project conception: CD, RP, FG, LB, and JD; Research project organization: CD, M-ÈR, AB, FB, and JD; Research project execution: AN, CD, M-ÈR, FB, and A-LL. Statistical analysis: AN and OL. Manuscript writing: AN, OL, and JD; Manuscript review and critique: AN, CD, OL, M-ÈR, AB, FB, RP, A-LL, FG, LB, and JD.

## Conflict of Interest Statement

The authors declare that the research was conducted in the absence of any commercial or financial relationships that could be construed as a potential conflict of interest.
