# Supplementary material for: A 12-Week Cycling Training Regimen Improves Gait and Executive Functions Concomitantly in People with Parkinson’s Disease
Source: Front Hum Neurosci. 2017 Jan 12;10:690. doi: 10.3389/fnhum.2016.00690 (PMC5226941; doi:10.3389/fnhum.2016.00690)
Supplement: Supplementary file 1 [file Data_Sheet_1.PDF]

Supplemental Material

Figure S1. AET-related changes in various domains

A.

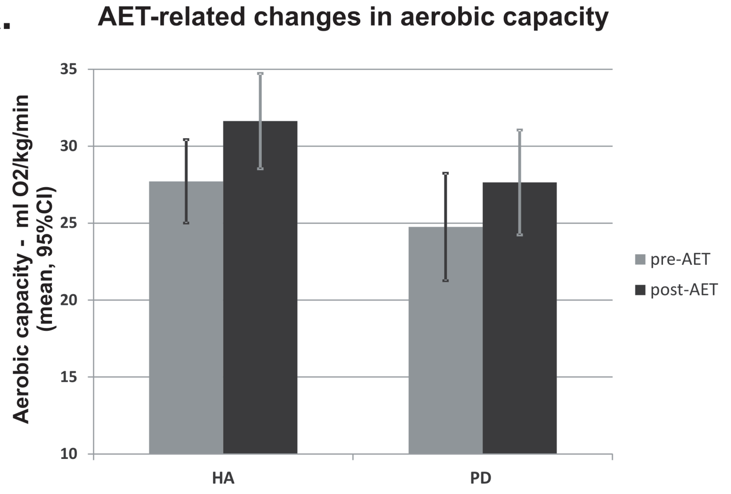

We observed significant AET-related changes in aerobic capacity in both healthy adults HA ($F_{1,35}$=15.19, p<0.001) and PD ($F_{1,35}$=9.98, p<0.003) groups.

B.

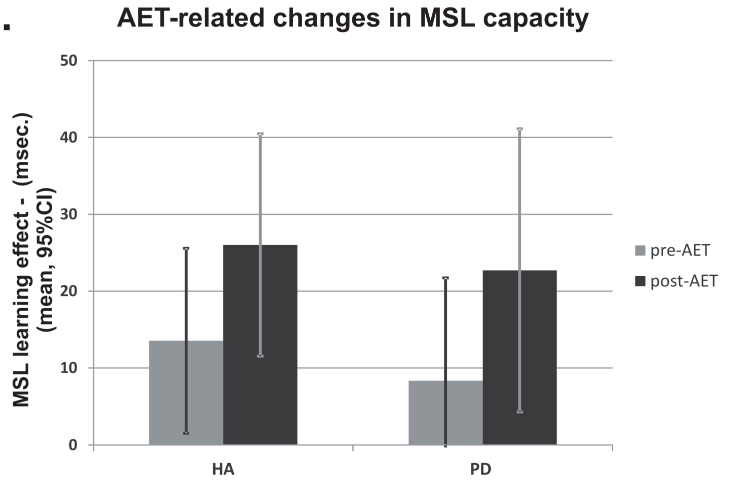

We observed marginally significant AET-related effect in MSL capacity only when both groups were combined ($F_{1,35}$=3.65, p=0.064). Nevertheless, there was a significant MSL effect (i.e. faster execution of sequential versus random movements) pre-AET in HC ($F_{1,35}$=4.06, p=0.05), but not in PD ($F_{1,35}$=1.43, p=0.24), whereas after training, both groups showed significant learning effects ($F_{1,35}$=7.13, p<0.05, for HC and $F_{1,35}$=8.88, p<0.005 for PD).

C.

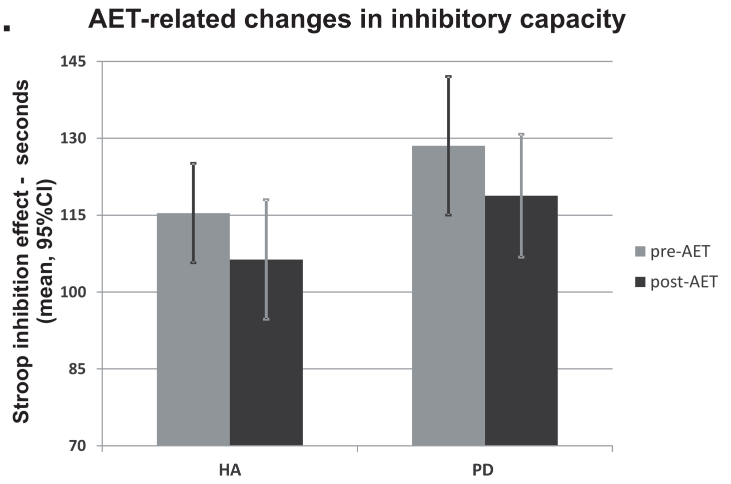

We observed significant AET-related changes in inhibitory capacity in both healthy adults HA ($F_{1,35}$=5.52, p<0.05) and PD ($F_{1,35}$=7.82, p<0.01) groups.

Legend: HA: healthy adults; PD: Parkinson's disease; AET: aerobic exercise training; ml: millilitres, kg: kilogram, min: minute; sec: seconds; MSL: motor sequence learning, difference between random and sequential block; msec: milliseconds
